# Postoperative Concurrent Chemoradiotherapy for Locally Advanced Thoracic Esophageal Squamous Cell Carcinoma: A Phase II Clinical Trial

**DOI:** 10.3389/fonc.2022.900443

**Published:** 2022-06-30

**Authors:** Hui Li, Dayong Gu, Mingyu Du, Guoren Zhou, Zhi Zhang, Jinjun Ye

**Affiliations:** ^1^ Department of Radiation Oncology, Affiliated Cancer Hospital of Nanjing Medical University, Jiangsu Cancer Hospital, Jiangsu Institute of Cancer Research, Nanjing, China; ^2^ Department of Oncology, Affiliated Cancer Hospital of Nanjing Medical University, Jiangsu Cancer Hospital, Jiangsu Institute of Cancer Research, Nanjing, China; ^3^ Department of Thoracic Surgery, Affiliated Cancer Hospital of Nanjing Medical University, Jiangsu Cancer Hospital, Jiangsu Institute of Cancer Research, Nanjing, China

**Keywords:** esophageal squamous cell carcinoma, IMRT, postoperative treatment, CTVs, cover partial regional lymph node areas

## Abstract

**Background:**

This study aims to investigate the efficacy and safety of postoperative intensity-modulated radiotherapy (IMRT) covering partial regional lymph node areas combined with chemotherapy for locally advanced thoracic esophageal squamous cell carcinoma patients.

**Methods:**

This was a single-center, single-arm phase II clinical trial that began in 2014. Patients who underwent radical transthoracic resection within 3 months and were histologically confirmed esophageal squamous cell carcinoma (pT3-4 or N+, M0 determined according to AJCC Guidelines, Edition 7) were recruited. Postoperative radiotherapy was performed with a total dose of 50.4Gy in 28 fractions using IMRT. Clinical target volumes (CTVs) included tumor bed, anastomosis, bilateral supraclavicular region, and superior mediastinal lymph nodes. Synchronous chemotherapy for 2 cycles (paclitaxel 150mg/m^2^, day1; Cisplatin 25mg/m^2^, day1-3; every 4 weeks), followed by 2 cycles of consolidation chemotherapy with the same regimen. The primary endpoint was the 2-year local control rate, and the secondary endpoints were overall survival (OS) and adverse events (AEs).

**Results:**

A total of 75 eligible patients were included from 2014 to 2017. The 2-year LRFS rate, as the primary endpoint, was 73.3%. The 1-year and 3-year OS rates were 88.0% and 68.0%, respectively. Local recurrence occurred in 13/75 (17.4%) patients, of which 2.7% (2/75) were extra-target lymph nodes. Grade 4 adverse events reported in this study included 10 cases (13.3%) of neutropenia, 1 case (1.3%) of anemia, and 2 cases (2.7%) of thrombocytopenia, without toxic-related deaths. Almost all (96%) patients completed the entire postoperative radiotherapy course, and 62 (82.7%) patients completed at least 2 cycles of chemotherapy.

**Conclusion:**

Postoperative IMRT (clinical target volume including tumor bed, anastomosis, bilateral supraclavicular region, and superior mediastinal lymph nodes) combined with synchronous chemotherapy in patients with locally advanced thoracic esophageal squamous cell carcinoma was well tolerated, with a high local control rate and a low probability of recurrence outside the irradiation field.

**Clinical Trial Registration:**

https://clinicaltrials.gov/ChiCTR1900022689.

## Introduction

Esophageal cancer (EC) is one of the most common malignant tumors in the world (1). It is also one of the 10 most common causes of cancer death in China (2). More than 95% of pathological types of esophageal cancer in China are squamous cell carcinoma, and thoracic esophageal squamous cell carcinoma accounts for more than 90% of all esophageal squamous cell carcinoma (3). Radical surgery is the main treatment for thoracic esophageal squamous cell carcinoma, but the 5-year survival rate of surgery alone rarely exceeds 30% (4), mainly due to local-regional recurrence and/or distant metastasis (5).In theory, postoperative adjuvant therapy can reduce recurrence rates and prolong survival by killing residual and subclinical lesions in patients with esophageal cancer.

In subgroup analyses of some Phase III randomized studies (6–8), postoperative radiotherapy did not improve efficacy. These studies were carried out relatively early and may be biased by different recruitment criteria, low-quality two-dimensional radiotherapy equipment, and inconsistent dose requirements. Nowadays, with the rapid development of radiation physics and medical imaging, IMRT technology has become the mainstream of modern radiotherapy technology due to its obvious dosimetry advantages. It achieves the consistency of a high-dose curve and radiotherapy target in three-dimensional space and is widely used in clinical work (9, 10).In recent years, more and more clinical trials and large sample retrospective studies have shown that postoperative radiotherapy can improve the local control rate and prolong the survival of patients with advanced esophageal cancer (T3/T4N+, M0) (11–13). Xiao et al. reported that in stage III esophageal patients (T4N0-1M0 or T3N1M0 determined by AJCC version 7), the 5-year survival rate in the postoperative radiotherapy group was higher than that in the surgery group (11, 12). However, it has been reported that the distant metastasis rate of postoperative prophylactic radiotherapy for esophageal cancer is relatively high, and in a recent Phase II study, the 3-year distant metastasis rate was over 40% (14). The results showed that postoperative prophylactic radiotherapy was only a local treatment method, which could not solve the potential risk of distant metastasis and recurrence of lesions outside the irradiation target. In addition, a subgroup analysis of phase III clinical studies in Japan (15) showed that postoperative chemotherapy can improve the efficacy of patients with positive lymph nodes. Therefore, in this study, postoperative prophylactic radiotherapy combined with chemotherapy was used to further control the distant metastasis rate of postoperative patients with esophageal cancer.

This single-center, single-arm, phase II clinical trial study aims to investigate the efficacy and safety of IMRT combined with chemotherapy in patients with locally advanced thoracic esophageal squamous cell carcinoma (T3-4 or N+, M0). Retrospective analysis (5) showed that the main recurrence sites after radical resection of esophageal cancer were bilateral supraclavicular region and superior mediastinal lymph nodes. Considering the protection of normal tissues, CTVs only included tumor bed, anastomosis, bilateral supraclavicular region, and superior mediastinal lymph nodes.

## Materials and Methods

### Study Design

This trial was a single-center single-arm, phase II clinical trial conducted from 2014 to 2017 at Jiangsu cancer hospital. Patients who were treated with radical transthoracic resection (with negative margins) within 3 months and histologically confirmed esophageal squamous cell carcinoma (pT3-4 or N+, M0 determined by the 7th edition of the AJCC guidelines) were qualified. None of the patients received preoperative or postoperative radiotherapy and/or chemotherapy prior to recruitment. No locoregional recurrent disease or distant metastases were found before postoperative radiotherapy. The inclusion criteria were as follows: 18 ≤ age ≤ 75 years; Eastern Cooperative Oncology Group (ECOG) performance status of 0 to 2; neutrophil count ≥ 2.0 × 10^9^/L; leukocyte count ≥ 4.0 × 10^9^/L; platelet count ≥100 × 10^9^/L; serum creatinine (Scr) level ≤ 1.5 upper limit of normal (ULN); and alanine aminotransferase (ALT) or aspartate transaminase (AST) ≤ 2.5 ULN. All participants provided written informed consent.

**Figure 1:**
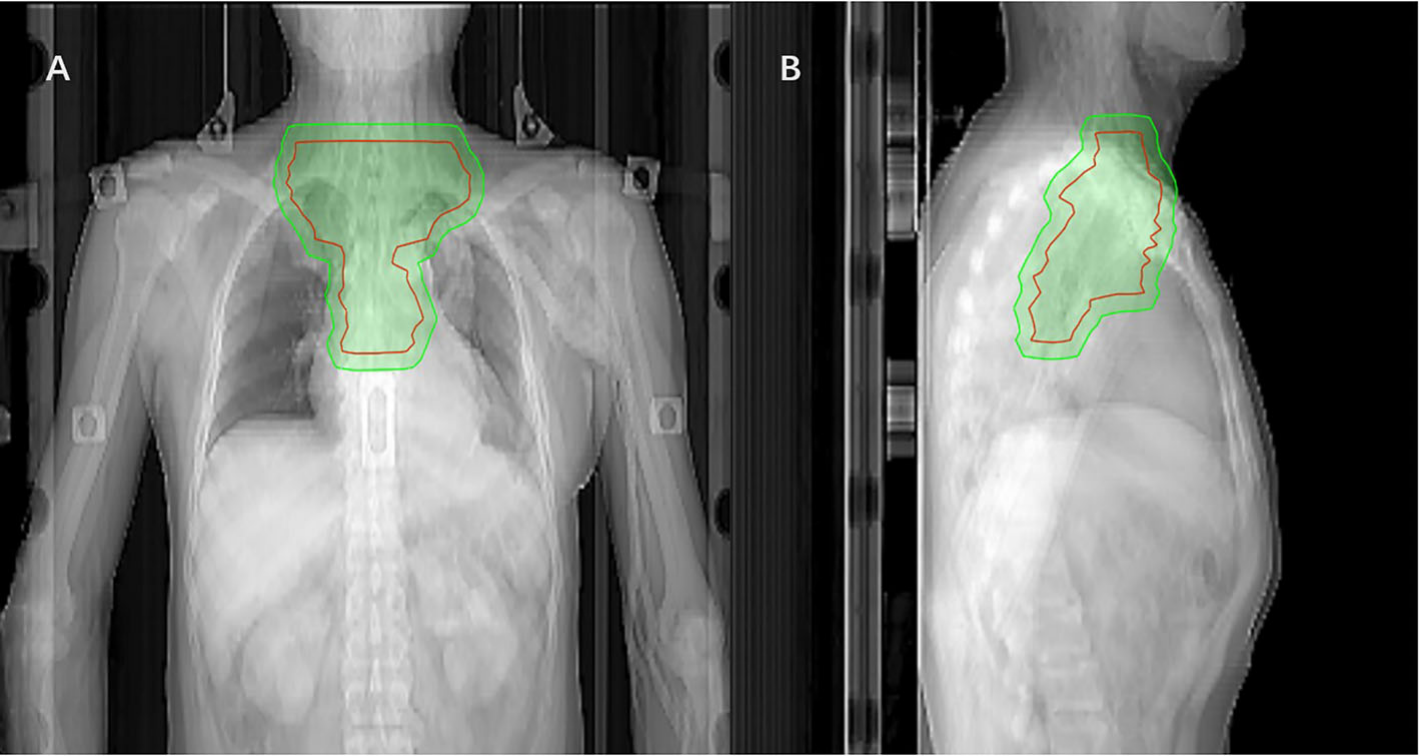
Coronal **(A)** and sagittal **(B)** views of the CTV (red mark) of postoperative radiotherapy using the IMRT(The green area is the PTV).

Radiotherapy was initiated within 3 months after surgery using 6-MeV photons delivered by a linear accelerator for a total dose of 50.4Gy in 28 fractions using IMRT. The CTVs included the tumor bed, anastomosis, bilateral supraclavicular region, and superior mediastinal lymph nodes (see **Figure 1** for details). The planned target volume (PTV) was externally placed 0.8cm around and above the CTVs, and the spinal cord side could be modified as appropriate to avoid high doses of spinal cord exposure. Dosimetry of tumor target area was mainly satisfied: The 95% isodose had to encompass the entire PTV. The maximum dose delivered to the PTV did not exceed the prescription dose by 10%. Dose limits for critical organs are shown in **Supplementary File 1**. The patients were followed-up at least once a week during the courses of postoperative treatment to monitor AEs. Radiotherapy was delayed when ANC < 1.0×10^9^/L (WBC < 2×10^9^/L while ANC was not available) or PLT < 50×10^12^/L. Radiation therapy can be suspended for up to 2 weeks, otherwise, it should be terminated in principle unless the investigator deems it necessary to continue radiation therapy.

Concurrent chemotherapy was conducted on the first day of radiotherapy: paclitaxel (150 mg/m^2^), continuous intravenous drip for 3 hours, day 1; cisplatin (25 mg/m^2^), i.v.drip., days 1 to 3. Cycles were duplicated every 4 weeks, for 4 courses altogether. For specific chemotherapy drug dose adjustment principles see **Supplementary File 2**.

The patients were followed up for local recurrence and distant metastasis every 3 months in the first year, every 4 months in the second year, and every 6 months within the third to fifth years after treatment. Imaging review mainly includes enhanced CT scan of the neck, chest, and abdomen, esophageal barium meal, and gastroscopy once a year.

### Outcomes

The primary endpoint was the 2-year local control rate of all enrolled patients. Secondary endpoints included toxicity and overall survival (OS). Toxicity was assessed according to the National Cancer Institute Standard for General Terminology of Adverse Events (AEs) (NCI-CTCAE 4.0). OS is defined as the time from the date of surgery to death. Disease-free survival (DFS) was defined as the time from the date of surgery to recurrence, metastasis, or death, whichever occurred first. Distant metastasis-free survival (DMFS) was defined as the time from the date of surgery to any recurrence beyond the primary tumor area and regional lymph nodes. Locoregional recurrence-free survival (LRFS) was defined as the time from the date of surgery until the date of recurrence in the esophageal stump or regional lymph nodes or death due to any reason.

### Statistical Analysis

In the CROSS clinical trial, the 2-year local recurrence rate of bilateral supraclavicular area, mediastinal lymph nodes, and anastomosis in patients with esophageal cancer in the surgical group was 34%, and the 2-year local recurrence rate of patients undergoing neoadjuvant chemoradiotherapy was 14% (16). Therefore, we expected a 20% improvement in the 2-year local recurrence rate in the postoperative chemoradiotherapy group. With a global alpha risk of 5%, power of 80%, and 10% patient loss, the necessary sample size was calculated. Categorical variables were descriptively analyzed by frequency and proportion. Median and inter-quartile range (IQR) were used to summarize continuous variables. Survival curves were plotted using the Kaplan-Meier method, with log-rank tests used to compare OS in subgroups, and hazard ratios (HR) were estimated using Cox regression models. SPSS 25.0 was used for data analyses.

## Results

### Clinical Characteristics

A total of 75 eligible patients were enrolled in this Phase II study between 2014 and 2017 (**Supplementary File 3**), and their baseline characteristics are listed in **Table 1**. The median age of enrolled patients was 62 years (IQR, 55-65 years), and the overwhelming majority were male (65 cases, 86.7%). The preoperative median tumor length was 4cm (IQR, 3-5cm), and most tumors were located in the middle thoracic region (66.7%). Two-field lymphadenectomy was performed in 40 patients (53.3%) with esophageal cancer, and the mean number of lymph node dissections was 12.4. In terms of tumor stage, stage IIa (44.0%) and IIIa (30.7%) were the most frequently examined.

**Table 1:** Patient Characteristics (N = 75).

Characteristics	N (%)
Age (years)	
Median (range)	62 (IQR 55-65)
Sex	
Male	65 (86.7)
Female	10 (13.3)
ECOG performance status	
0	65 (86.7)
1	10 (13.3)
Tumor stage (AJCC, 7th edition)	
IIa	33 (44.0)
IIb	8 (10.7)
IIIa	23 (30.7)
IIIb	6 (8.0)
IIIc	5 (6.7)
T phase	
T1	4 (5.3)
T2	8 (10.7)
T3	60 (80.0)
T4a	3 (4.0)
N phase	
N0	34 (45.3)
N1	28 (37.3)
N2	9 (12.0)
N3	4 (5.3)
Tumor location	
Upper (<25cm)	5 (6.7)
Middle (25-30cm)	50 (66.7)
Lower (>30cm)	19 (25.3)
Multi-primary	1 (1.3)
Tumor length, cm	
Median (range)	4 (IQR, 3-5)
<5	45 (60.0)
≥5	30 (60.0)
Lymphadenectomy	
Two-field	40 (53.3)
Three-field	35 (46.7)

### Treatment

Postoperative radiotherapy was completed in almost all patients, with a completion rate of 96% (72/75). Three patients terminated radiotherapy due to radiation pneumonia, asthma attack, and arrhythmia, respectively. In terms of chemotherapy, 62 patients (82.7%) completed at least 2 cycles of treatment, 36% (27/75) of patients underwent at least 3 cycles of chemotherapy, and 22.7% (17/75) received all 4 cycles. Paclitaxel and cisplatin were reduced by 20% and 25% in 14 patients (18.7%) because of toxicity during treatment.

### Survival and Treatment Failure Pattern

**Figure 2:**
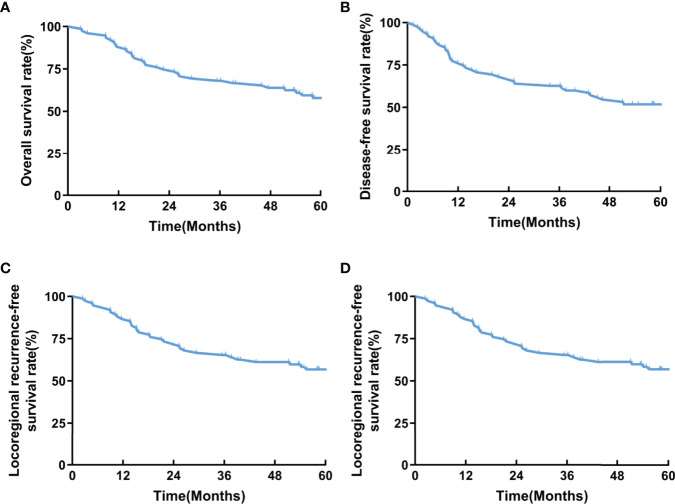
**(A)** OS, **(B)** DFS, **(C)** LRFS, **(D)** DMFS of the enrolled patients.

A total of 75 patients were recruited from 2014 to 2017 and followed up to December 2021 with a median follow-up duration of 58.2 months (IQR, 22.6-77.3 months). Thirty-six patients died during the follow-up. The 2-year LRFS rate, as the primary endpoint, was 73.3%. The 1-year and 3-year LRFS rates were 86.7% and 65.3%, respectively. The 1-year and 3-year OS rates were 88.0% and 68.0%, respectively. The 1-year and 3-year DFS rates were 76.0% and 62.7%, respectively. The 1-year and 3-year DMFS rates were 77.3% and 65.3%, respectively (**Figure 2**).

**Figure 3:**
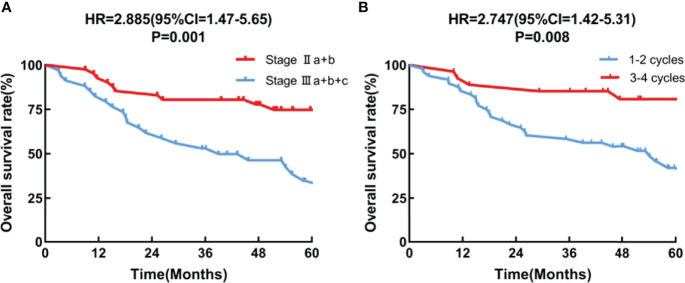
Overall survival curves of subgroups **(A)** pathologic stage, **(B)** chemotherapy cycles.

Multivariate Cox regression analysis showed that the tumor stage (HR = 2.885, 95*CI* = 1.47-5.65; *P* = 0.001), and the number of chemotherapy cycles (HR = 2.747, 95*CI* =1.42-5.31;*P* = 0.008) were independent prognostic factors (**Figure 3**).

Further analysis (**Table 2**) of the modes of initial treatment failure showed that 50.6% (38/75) patients had recurrence or distant metastasis, of which distant metastasis was the dominant mode of failure. Hematogenous recurrence occurred in 21 (28%) patients. The liver was the dominant organ of metastasis, followed by the lung and bone. Among the patients with local recurrence, most (11/75,14.7) recurred in the irradiation field, and 2 cases (2.7%) recurred in the celiac trunk lymph nodes. In this study, a second primary tumor (all more than 3 years after surgery) was observed in 5 patients, and 4 of these patients died of the second primary tumor. After adjusting for age, sex, tumor stage, and family history of cancer, multivariate analysis showed that patients younger than 62 years of age (HR 1.6) had a higher risk of the second primary malignancy. However, given the small sample size, the results should be treated with caution.

**Table 2:** Patterns of First Treatment Failure $.

First Failure	N (%) *
No Failure	37 (49.3%)
Loco-regional Failure (In field)	11 (14.7%)
Anastomosis	6 (8.0%)
Supraclavicular Lymph Node	1 (1.3%)
Mediastinal Lymph Node	4 (5.3%)
Loco-regional Failure (Out of field)	2 (2.7%)
Celiac Lymph Node	2 (2.7%)
Distant Metastasis	21 (28%)
Lung	8 (10.7%)
Liver	9 (12.0%)
Bone	4 (5.3%)
Pleura	3 (4.0%)
Stomach	1 (1.3%)
Secondary primary tumor	5 (6.7%)
Base of tongue	2 (2.7%)
Lung	1 (1.3%)
pancreas	2 (2.7%)

$Concurrent recurrence is defined as different recurrences within 2 months.

*There will be overlapping of patients in various recurrence situations, but the denominator is 75 when we calculating the ratio.

### Toxicity

All acute AEs reported are listed in **Table 3**, and the incidence of grade 3 or above AEs was 43.9%, mainly hematologic adverse events. Grade 4 hematologic AEs included neutropenia in 10 cases (13.3%), anemia in 1 case (1.3%), and thrombocytopenia in 2 cases (2.7%). In terms of non-hematologic adverse reactions, except for one case (1.3%) of grade 3 radiation pneumonia, the rest were grade 1-2, mainly radiation injury. None of the patients died from acute adverse reactions.

**Table 3:** Acute Treatment Toxicity.

Toxicity	N (%)			
	Grade 1	Grade 2	Grade 3	Grade 4
Hematological toxicity				
Neutropenia	15 (20.0)	21 (28.0)	18 (24.0)	10 (13.3)
Anemia	17 (22.7)	2 (2.7)	1 (1.3)	1 (1.3)
Thrombocytopenia	12 (16.0)	5 (6.7)	0	2 (2.7)
Nonhematological toxicity				
Fever	2 (2.7)	0	0	0
Neurotoxicity	3 (4.0)	0	0	0
Esophagitis	55 (73.3)	4 (5.3)	0	0
Pneumonitis	43 (57.3)	5 (6.7)	1 (1.3)	0
Dermatitis	37 (49.3)	5 (6.7)	0	0
Bloating and acid return	12 (16.0)	0	0	0

## Discussion

This phase II clinical trial was launched to explore the safety and efficacy of postoperative chemoradiotherapy in patients with locally advanced thoracic esophageal squamous cell carcinoma. Compared with previous studies, IMRT was used in this study, and CTVs did not cover all regional lymph nodes. The consistency of prescription dose and target volume was strictly evaluated before radiotherapy.

Currently, it is generally accepted internationally that esophageal cancer patients with a high risk of local recurrence (pT3-4 or N+ or resection margin +) need to receive adjuvant therapy after surgery (17). Zhang et al. (13) hold the opinion that in esophageal cancer patients with postoperative pathological stage IIb-III, the 5-year OS rate in the surgery + IMRT group was superior to that in the surgery-alone group (32.7% vs. 27%). In our study, the survival efficacy was further improved by the addition of concurrent chemotherapy. In Adelstein et al.’s study (18), the median time of freedom from recurrence was 48 months and the median overall survival is 53 months. Here, the median DFS and OS were 53.4 and 74.5 months, respectively, which were superior to those reported by Adelstein et al. Nonetheless, the 2-year local recurrence rate was higher than that in the CROSS study (16), which was used to calculate the sample size. We considered that adenocarcinoma accounted for 75% of patients enrolled in the CROSS trial, which may have resulted in these differences. In addition, by analyzing the results of this study, it was found that distant metastasis (mainly hematogenous metastasis) occurred in 28% of the 75 enrolled patients, which was similar to the failure mode of previous postoperative radiotherapy studies (25.7%) (14). However, the 3-year distant metastasis rate in this study was significantly reduced (34.7%vs.47.8%), indicating that postoperative adjuvant radiotherapy combined with chemotherapy can further delay the distant progression of patients.

Severe bone marrow suppression is rare in patients with esophageal cancer treated with radiotherapy alone. The incidence of grade 3 and above neutropenia in this study was 37.3%, which was similar to previous studies on postoperative chemoradiotherapy (18, 19). It is proved that postoperative patients with esophageal squamous cell carcinoma can tolerate the toxicity of IMRT combined with synchronous chemotherapy. The occurrence of hematological toxicity reminds us that during the postoperative treatment of esophageal cancer, especially for patients who have received too many courses of chemotherapy, we should pay attention to the detection of blood indicators and deal with them in advance to prevent severe myelosuppression.

At present, there is still no conclusion about the CTVs of prophylactic irradiation after radical resection of esophageal cancer, considering which may be related to the pattern and prevalence of lymphatic spread in thoracic esophageal carcinoma (20). In the radical resection of esophageal cancer, the left gastric and paracardial lymph nodes are easy to be thoroughly dissected. Esophageal cancer resection through the left thoracic approach is more likely to be blocked by the aortic arch and the ascending branch of the left subclavian artery. When the trachea, the adjacent areas of the bilateral recurrent laryngeal nerves, and the surrounding adipose tissue are completely removed, it is easy to cause residual subclinical lesions in the upper mediastinal lymph nodes after surgery, which will become the root cause of future recurrence. The traditional extensive CTV is bound to cause radiation injury, anastomotic stenosis, and other complications, so the safety and feasibility of which are still debatable. Moreover, some studies have found that radiation-induced side effects are more severe in the lower mediastinal region and upper abdominal region (12, 21). During the follow-up period of this study, 2 patients (2.7%) were tracked to have recurrence outside the irradiation target area (abdominal lymph node), which was similar to other literature reports (5, 20, 22), indicating that the risk of recurrence outside CTVs did not increase. But interesting to note: that fewer patients (25.3%) with lower thoracic esophageal cancer were enrolled in this study, so this conclusion should be treated with caution.

In addition, Chen et al. (21) believed that the number of cycles of postoperative chemotherapy for esophageal cancer was an independent factor of prognosis, and the OS of patients receiving 2-4 cycles of postoperative chemotherapy was significantly better than that of patients receiving 1 cycle (*p* = 0.001). Grade 3-4 hematologic AEs slightly increased due to maintenance chemotherapy but remained manageable. Moreover, OS and DMFS were also relatively prolonged. In our trial, we discovered that patients who underwent 3-4 chemotherapy cycles had a significantly higher OS rate than did those who underwent 1-2 cycles (*p* = 0.008). However, the OS rates were similar among patients who underwent 3 or 4 chemotherapy cycles (*p* = 0.46, **Supplementary File 4**). Although 36% (27/75) of patients completed at least 3 cycles of chemotherapy in this study, this regimen remained difficult for patients. Recovery after esophagogastric resection is slow and may impede the use of postoperative chemoradiotherapy. Despite better nutritional support, the overall physical state is often reduced after surgery, potentially interfering with overall treatment tolerance. Furthermore, the impact of the altered postoperative blood supply on the delivery of chemotherapy and oxygenation of the tumor bed must be taken into account.

Several limitations should be considered when interpreting our findings. First, this was a single-arm study lacking a control group. Further research on this approach, particularly in comparison with preoperative chemoradiotherapy, is justified. Second, some recruited patients had a history of surgery at other hospitals, and their medical history was not sufficiently detailed.

## Conclusions

In general, postoperative IMRT combined with chemotherapy for locally advanced thoracic esophageal squamous cell carcinoma is safe and effective. Compared with the traditional extensive CTVs, the CTVs of our study minified the inferior mediastinum, left gastric, and celiac trunk lymph nodes, and the results suggested that it was feasible, providing a basis for further phase III studies.

## Data Availability Statement

The datasets presented in this study can be found in online repositories. The names of the repository/repositories and accession number(s) can be found in the article/**Supplementary Material**.

## Ethics Statement

The studies involving human participants were reviewed and approved by Ethics Committee of Cancer Hospital Affiliated to Nanjing Medical University. The patients/participants provided their written informed consent to participate in this study.

## Author Contributions

HL and DG analyzed the data and were major contributors to writing the manuscript. JY was a major contributor to the trial design and the enrollment of patients. MD, GZ, and ZZ are responsible for the enrollment, efficacy, and safety records of the patients. All authors contributed to the article and approved the submitted version.

## Funding

This work was supported by Six Talent Peaks Project in Jiangsu Province (CN) [grant numbers: TD-SWYY-007]; Wu Jieping Medical Foundation Project (CN) [grant numbers: 320.6750.19194-60].

## Conflict of Interest

The authors declare that the research was conducted in the absence of any commercial or financial relationships that could be construed as a potential conflict of interest.

## Publisher’s Note

All claims expressed in this article are solely those of the authors and do not necessarily represent those of their affiliated organizations, or those of the publisher, the editors and the reviewers. Any product that may be evaluated in this article, or claim that may be made by its manufacturer, is not guaranteed or endorsed by the publisher.
