# Supplementary material for: Postoperative Concurrent Chemoradiotherapy for Locally Advanced Thoracic Esophageal Squamous Cell Carcinoma: A Phase II Clinical Trial
Source: Front Oncol. 2022 Jun 30;12:900443. doi: 10.3389/fonc.2022.900443 (PMC9280634; doi:10.3389/fonc.2022.900443)
Supplement: Supplementary file 1 [file DataSheet_1.docx]

**supplemental file 1**

Inclusion criteria

1) Sign the informed consent;

2) Age 18-75 years old;

3) Histologically confirmed esophageal squamous cell carcinoma, initial treatment (without surgery/radiotherapy/chemotherapy and targeted therapy);

4) Locally advanced: T2-4N0M0, TxN1M0 and TxNxM1a (AJCC 6th edition esophageal cancer staging system)

5) Non-pregnant or breastfeeding women, fertile men or women willing to take contraceptive measures in the trial;

6) No serious hematopoietic function, heart, lung, liver, kidney dysfunction and immune deficiency;

7) Normal organ and bone marrow function, defined as follows: white blood cell count ≥ 4,000/µL, hemoglobin ≥ 9 g/dL (without blood transfusion), neutrophil count ≥ 2,100/µL, platelet count ≥ 100,000/µL, total bilirubin < 1.5 times the upper limit of normal, AST (SGOT)/ALT (SGPT) ≤ 2.5 times the upper limit of normal, and serum creatinine ≤ 1.5 times the upper limit of normal.

8) Physical condition score ECOG 0-2;

9) Weight loss ≤10% within 6 months before radiotherapy;

10) Expected survival period > 3 months;

11) There is no primary tumor in other parts before treatment, or there is a primary tumor in other parts but it has been controlled for more than 5 years;

Exclusion Criteria

1) The esophagus is completely obstructed and cannot eat liquid, and the esophagus has deep ulcer or perforation or hematemesis;

2) The second primary tumor (cured non-malignant melanoma of the skin, papillary thyroid carcinoma, stage I seminoma, carcinoma in situ of the cervix, or other "curable" tumors) survives tumor-free for more than 3 years after treatment except);

3) Have used other experimental drugs or participated in other clinical trials 30 days before randomization;

4) Pregnant, breastfeeding or fertile but not taking contraceptive measures;

5) Drug addiction and other adverse drug addiction, long-term alcohol addiction, and AIDS patients;

6) Myocardial infarction time ≤ 6 months, myocardial infarction time > 6 months, myocardial thallium angiography is required to show no myocardial ischemia and the cardiologist agrees to chemotherapy can be included in the study;

7) Uncontrollable epileptic seizures, or loss of insight due to mental illness;

8) Those with a history of severe allergies or specific constitutions;

9) The researchers believe that it is not suitable to participate in this trial;

10) Have received radiation therapy in the area of this scheduled radiation therapy.

Termination criteria

Patients should be discontinued and followed until death in the following cases:

1) All treatments have been completed;

2) Cannot be treated according to the requirements of the research protocol;

3) Disease progression (PD) in the trial;

4) Severe allergic reactions or serious adverse events occur;

5) Subjects request to withdraw and withdraw informed consent;

6) The patient is pregnant or not using adequate contraceptive measures;

7) After the second dose adjustment, the subject still cannot tolerate the toxicity;

8) The researcher deems it necessary to withdraw from the study.

**supplemental file 2**

**Dose adjustment principle**

All patients were given the planned chemotherapy dose in principle, and the dose could be adjusted if necessary according to the most serious hematological or other toxicity. Any patient requiring a dose reduction will continue to receive the reduced dose for subsequent treatment cycles. If a patient presents with multiple toxicities and the principles of dose adjustment differ from one another, the lowest dose is selected. If ANC ≤ 1.5 x 109/L and platelets <100 x 109/L on day 1 of the course of treatment, the medication was suspended for one week. Except for alopecia, nausea and vomiting, other non-hematological toxicities of grade II and above should be recovered to grade 0 to 1 before the next course of treatment can be performed. Recovery of hematologic and non-hematologic toxicities can be delayed up to 14 days. A maximum of two decrements are allowed. Dose reductions should be based on the grade of the most severe toxicity from the previous course of treatment. Cisplatin and carboplatin were adjusted to 75% of the original dose for the first time, and to 50% of the original dose for the second time. Paclitaxel was adjusted to 80% of the original dose for the first time, and adjusted to 60% of the original dose for the second time, and the dose adjustment scheme was adjusted according to the following.

1. Paclitaxel and DDP were both reduced: Grade III/IV neutropenia accompanied (≥38.3°C or 38.0°C for 1 hour). Grade IV neutropenia persisting for ≥7 days; other investigators believe that dose reduction is warranted.
2. Cisplatin dose reduction: Adjust the dose of DDP according to the renal function. When 45ml/min≤ creatinine clearance (CCI) <60ml/min, the DDP dose is reduced by 25%; when 30ml/min ≤ creatinine clearance (CCI) <45ml/min, the DDP dose is reduced by 50%; when creatinine clearance (CCI) <30ml/min, the medication should be suspended. The next cycle of treatment cannot be started until recovery to level 0-1.
3. Paclitaxel dose reduction: For severe peripheral neuropathy, the dose should be adjusted or discontinued according to the total dose principle.

Note: DDP is only decremented once when both 1 and 2 are present; Paclitaxel was reduced only once when both 1 and 3 occurred.

**supplemental file 3**


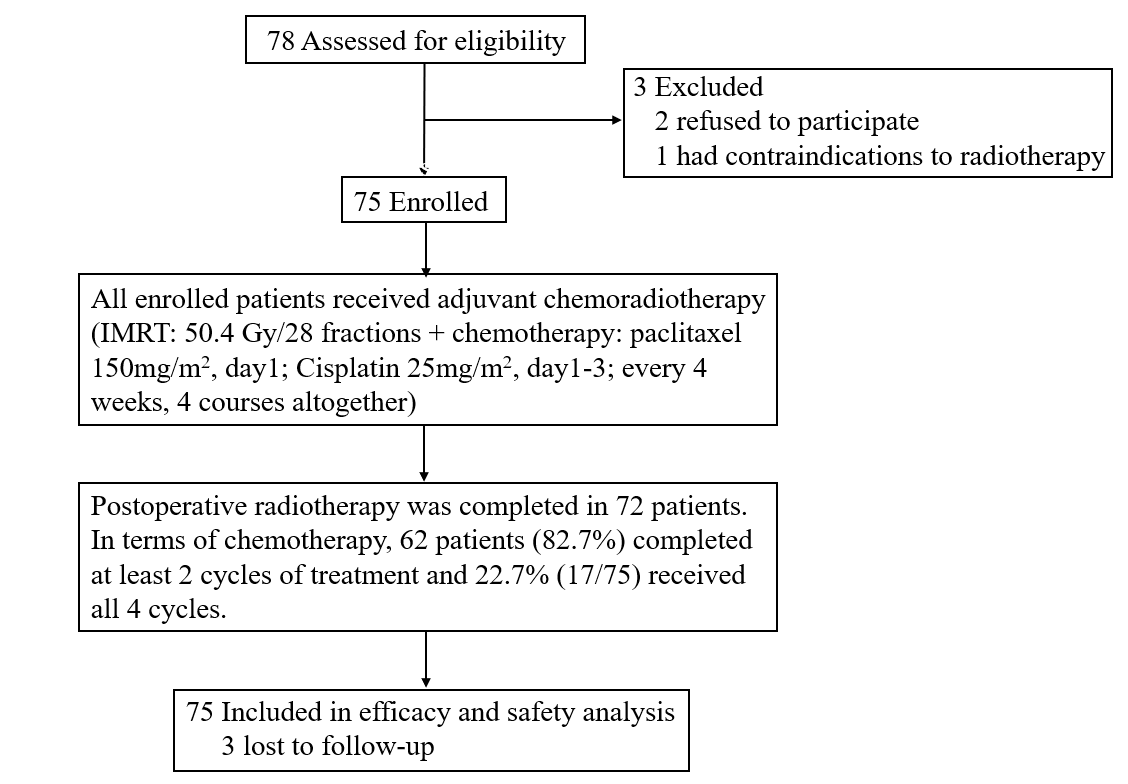


**supplemental file 4**


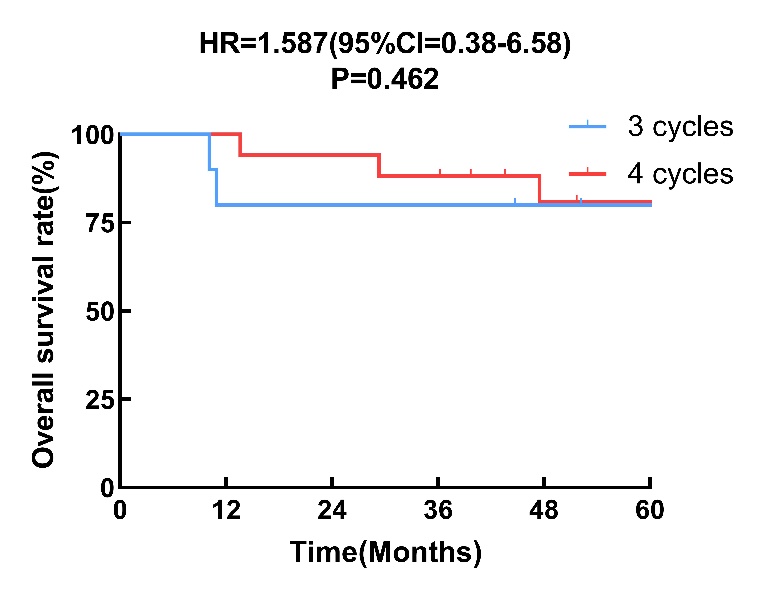


Kaplan–Meier curve of overall survival for patients receiving 3 cycles of chemotherapy versus 4 cycles.
